# Something Small That Matters†

**DOI:** 10.4269/ajtmh.19-0888

**Published:** 2019-12-19

**Authors:** Chandy C. John

**Affiliations:** Ryan White Center for Pediatric Infectious Diseases and Global Health, Indiana University School of Medicine, Indianapolis, Indiana

Thank you, Pat, for that wonderful introduction and for all that you’ve done for me and for this Society as a leader and guide.

It has been a tremendous honor to serve as president of this great society. As I end my term with this address, I decided to take a bit of a risk. This address will be different from all preceding presidential addresses. For one thing, sure to be welcomed by all, it will be the shortest address on record. For another, it will take a different approach to the form of a presidential address.

**Figure:**
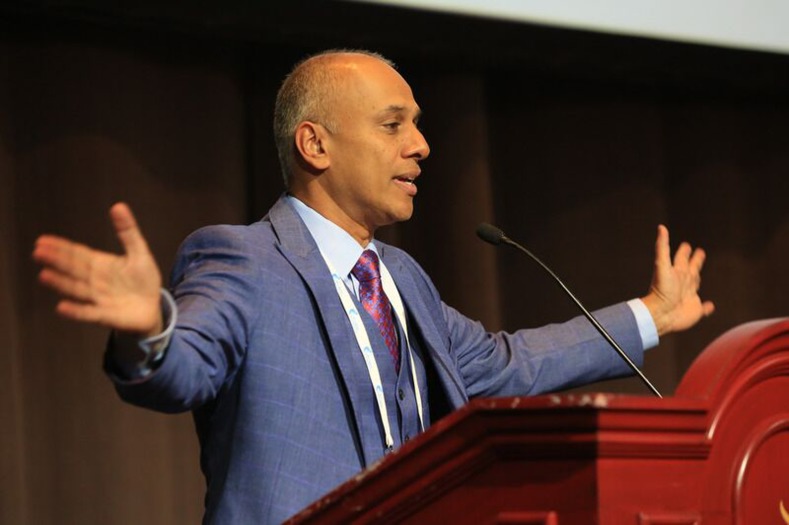
President Chandy C. John, MD, MS, FASTMH, delivers his President’s Address during the 2019 Annual Meeting in National Harbor, MD.

I’ve been thinking a lot about where we are heading as a Society, big S, and society, small s, and it seems to me that we are at a crossroads right now, where what we do and what we stand for will make a great difference for the future. In the world today, science is routinely attacked, and demagogues rally around the promise of demonizing the other. In such a world, our work in doing, communicating, and putting into public policy good science that save lives, and our work in reiterating the basics of human rights for all and the interconnected nature of our world, which precludes division into an “us” and “them,” is more important than ever.

I titled my talk “Something small that matters” because I want us to think about these issues and this vision in terms of the big changes we can and must make, but also in terms of the small things we can do everyday that matter. The further I’ve gotten into my medical and research career, the more I’ve realized that most of the time, the work our group does will contribute one small piece to the understanding of disease or epidemiology or pathogenesis, and it will have its little part in the bigger picture, but it will only be just one small part. But science is mostly a process of increments, one bit of learning building on another. So our small project—or yours—may not change the world, but if it adds knowledge, it’s worth doing. And someday, you may make that big breakthrough, but if your whole career is a career of small moments of something new, that’s fine too. It’s something small, but it matters.

I think, as a physician-scientist, I have been able to see this most clearly in my dual roles. I went into research because I wanted to be part of the solution to the problems I was seeing in my patients and, particularly, in patients I took care of in Africa and Asia when I worked there. The huge burden of malaria I saw in Nigeria cemented my decision to make work in this area my life goal. And doing the research has been hugely rewarding, one of the great joys of my life. But we haven’t solved the problem yet. There’s been a lot of progress in understanding malaria and in reducing malaria cases and deaths, but our work has been only a small part of that. We hope for bigger outcomes, such as success in eliminating malaria in the highland areas of Kenya, where we’ve worked for the past 23 years, or of reducing malaria deaths and neurodevelopmental complications in our study areas in Uganda and beyond. But those successes are still some years away, if indeed they occur at all. My work of taking care of patients, the one on one of seeing that individual child getting better, because of the work that doctors and nurses and pharmacists do, helps me see the value in the smaller scale. Just like taking care of that single child has its own reward; doing those small studies; working with smart students, residents and fellows; and learning new things that don’t have immediate translation, yet help us understand some aspect of malaria better—each of these has its worth. We never stop aiming big, but everyday we can do that something small that matters.

I see this, too, in the way the center I work in at Indiana University was developed. I’m the director of the Ryan White Center for Pediatric Infectious Diseases and Global Health. People often ask me about the rich donor who endowed our center, and I’m always happy to reply that the rich donor is thousands of college students, my beloved Indiana University dance marathoners. Over the years, these college students have raised millions of dollars, first for our center, to do clinical work and research that prevents and treats infectious diseases in children, and now for the whole department of pediatrics, to improve the health of children in Indiana and around the world. The dance marathons started as a tribute to Ryan White, an Indiana hero who was the first child in the United States to make people publicly aware of the costs of HIV in childhood. He and his mother, Jeanne White, fought for a normal life for every person with HIV. And his life, work, and message directly led to funding that has improved the lives of millions around the world with HIV. One little kid in Indiana did that. Ryan White’s doctor was the founder of our division, Marty Kleiman. Ryan’s friend, Jill Stewart, talked to Dr. Kleiman about how best to honor Ryan’s legacy and started the Indiana University Dance Marathon in his honor, with a few of her college friends, to raise funds to do research in pediatric infectious diseases and to prevent and treat infections such as HIV. That first year, it raised $25,000. This year, it raised more than $4 million for our department of pediatrics. And this was all from college students who want to make a difference in their world. That growth from a small operation to a powerhouse fundraiser is wonderful and powerful, but if it had only been the $25,000 that was raised, that would have been important all by itself. It was something small that mattered.

As I think about our Society and the work we aim to address, I look at things in the same way—the big goals and the small steps that get us there. The initiatives I focused on, in addition to the ongoing and primary initiative of great science and clinical work that serves the underserved, are increasing engagement and retention of trainees and low- and middle-income members, moving toward certification of a medical specialty in global medicine and increasing funding to support all of this. A president serves for 1 year. These goals will be worked on over many years.

We’ve made a start on bigger goals with committees for international members, trainee members and global medicine certification, and with the hire of a development officer to increase fundraising. The committees have proposed specific first step initiatives that the board has included in our strategic plan, and many more will come over the next several years. I’m confident that these will make our Society stronger and better in these key areas. But the presidents’ challenge was a way to get a concrete start on three of these key initiatives right in this year of my presidency. The president’s challenge will support additional 100 trainee travel awards over the next 5 years, and 75 of those awards will go to trainees from low- and middle-income countries. So, it’s addressing trainee and LMIC member engagement and retention, and it’s fostering a culture of giving to our Society by its members. Thanks to your generosity, and in particular the generosity of the board and our past and incoming presidents, we have crossed the $90,000 mark, 60% of the way to our goal of $150,000. I encourage you to continue giving so that we reach the mark this year, to fund awards through 2023. It’s a start, and an important one, toward our bigger goal. It matters.

In the spirit of small things that matter, I want to shift now to the unorthodox component of this address that I warned you about at the start.

My first love has always been the arts. My life has been enriched immeasurably by my partner, Andrew Hisey, and one of the many ways he’s enriched my life is through his music. He’s a classical pianist, and the glorious music he plays has been the soundtrack of my life for the past 26 years. Sadly, I don’t share his exceptional musical gifts, so the surprise for this address is not that we’re hauling in a piano for me to do a recital. My gifts in the arts, such as they are, have been in the area of writing, and one form of art I particularly love is poetry. Poetry is in some ways a good analogue to scientific writing because in both, one strives to provide the findings in the most concise and accurate way possible. The difference is that science typically aims to be dry and “just the facts.” We judge the quality of science based on what the data show and the implications of the study findings. Poetry, in contrast, is all about emotion. It’ s a terrible way to communicate science—hence, the lack of science manuscripts in poetry form—but it can be a highly effective way to communicate ideas and moments and the meaning behind the things we do.

When I think about a presidential address, and what it leaves for the listener after the address is over and how it might read to a quizzical Society member 10 or 20 years down the road, I think that the images provided by poetry might best give a sense of the issues we face and what we need to do to address them.

With that in mind, I’ve made the radical decision to offer the presidential address as a series of 10 poems that relate to key issues we face as a Society. I decided to focus on the really big picture: who we are, who we serve, and how and why we serve them. I’ll give a brief introduction to each poem, to set them in context. And I’m prepared afterward to bear the “slings and arrows” of outraged Society members. Make of the poems what you will. I hope they provide food for thought and help us all to consider our work in a different way. But maybe they’ll just crash and burn. You can tell me at the end—and I’m sure you will.

I want to start by considering who we serve. We serve the poor, the underserved, and the vulnerable. We don’t talk a lot about this, but it underlies everything we do. Our keynote speakers, the Bangs, and our distinguished guest speaker, Francis Collins, brought this idea to the forefront. With that mind, two poems: the first is called, “To the emaciated boy with the skull fracture.”

**To the emaciated boy with the skull fracture**

To the emaciated boy with the skull fracture

Your smile undid me

At once, I was not a doctor

Deducing the cause of your fever

I was instead witness to a sweet, sad miracle

A boy whose defense was his wall of solitude

Letting joy peep out like a shy duckling

In that quiet moment when smile met smile

I want to move on from that poem to a connected one, about who we serve and who leads us. To be a great society, we have to grow and cherish our future leaders, and those we serve must be among our leaders.

This poem is called “Mighty, brilliant, ebony and cinnamon girls.”

**Mighty brilliant ebony and cinnamon girls**

Mama

I see the way you hold your baby girl

She contains multitudes

Let us protect her from the lust of

Mosquitoes for her blood

Bacteria for her strength

Viruses for her defenses

Parasites for her power

Bitter men for her innocence

Rapacious men for her wealth

It is her wealth they squander

Her future they lay waste

Her identity they try to erase

Mamas

Your mighty, brilliant, ebony and cinnamon girls

Let us nourish them

Let them nourish us

I’ll move on to two poems that consider how we do and what we do. The first, called “Malaria,” is about the wonder and passion of science, and the second, “I see you,” is about how excellence and integrity in science are a commitment to those we serve.

**Malaria**

I will decode the spiky messages

That trapped my mother

I will deflect the probing fingers

That snared my father

I will defuse the hidden landmines

That maimed my brothers

I will expose each precious weapon

Until you have nothing left

This is a blood battle

**I see you**

I see you

Detecting coding errors

Inspecting miscalculations

Eyeing dilution problems

We’ll redo the experiment

We’ll correct the calculations

We will not say a word until we know

Every detail is right

Ugandan child

I am examining your life

And you are examining me

I have no room for error

Except, inevitably, in medicine and science, we all do make errors. And we don’t talk about them. And we don’t talk about the reality that our work in even small things can be disappointing and that life in science and medicine often feels like a long string of failures, broken intermittently, we hope, by a few successes. On the topic of who we are, I want to talk about facing failure honestly.

I’m the president of ASTMH, and I’m sad to report my inbox still features plenty of rejection letters. And this is true of scientists of much greater stature than me. We don’t talk about it enough. So, I thought it might be cathartic, especially for students and postdocs, to hear the next poem.

It’s called “Rejection letters.”

**Rejection letters**

Your font was too small

Your font was too big

You used the wrong font

Your conclusions are unsound

Your conclusions are sound, but don’t matter

Your conclusions are sound, and do matter, but we’re ignoring reviewers today

Your findings are different from the other study

Your findings are the same as the other study

Your findings lack impact

Your findings have impact, but not enough

Your findings have enough impact, but we know the other guy

Your findings might have been important but

Since you pointed out the flaws, we’re agreeing with them

Well, honest to God

What were you thinking?

My mom and dad always believed in my potential, and that buffered a lot of the rejection along the way, and it still does. I learned from them how important it is, no matter how big or critical the task at hand, to take the time to make people feel welcomed and cared for. I’ve learned that as well from Francis Collins, Jim Kazura, Cindy Howard, Pat Walker, Janet Gilsdorf, Carol Kauffman, and many other mentors. And that’s something I’ve valued in this Society from the start: the sense that I’m welcomed, that we welcome everyone, and whether you’re a Nobel prize winner or a new student, you are welcome and valued. The sense of welcome can come from something big and bold, like the sign announcing the conference that shows many different types of people, or a little gesture like a smile or a greeting. My mom is a master at doing things big and small to make people feel welcome and accepted. This poem in honor of that very needed skill is called “The small things.”

**The small things**

This was the ritual:

Mom would kiss her two little boys goodbye

Pull her sari into the car

And start her drive to the hospital

Sunil and I would run frantically behind

Until she stopped, mid-driveway

Got out of the car

Kissed us tenderly on the head with those soft, soft lips

And then said goodbye, again

It must have gotten old

Having to stop each time

As she was rushing to the hospital to see patients

But she did it

Every single day

Sometimes

The small things matter

So much

When I think of who we are, I think of those we’ve learned from as mentors. When you move into a mentor role yourself, you realize all those things your mentors had to deal with that you didn’t know about, and how calmly they did it, protecting you from the stress by absorbing it themselves. As I’ve understood their vulnerability, my respect for them increased. The next poem is for my dad, but also for all of those who’ve guided us along the way.

**The day I learned my Dad was Superman**

When I was in fifth grade,

A friend pointed out my Dad's bowed legs

I ignored the friend for days

I was Mama’s boy

I studied Dad from two steps away

Some days, I caught a glimpse of what was behind

Doctor Dad

Other days, he was a wall

Dad was earnest in a way that made me cringe

Sometimes

Or stern and cold, which

Also made me cringe

A little thing could draw out the fire inside

Often enough to make you careful

Rare enough to surprise you

Dad was not tough, his jaw was not square

He was small, thin, friendly, aloof

Quiet, loquacious, cautious, eager

He insisted, always, on being himself

He was there, for us to be proud of

Or not, he would not change

I was on my own, living far away from him

When Mom called to tell me about the heart attack

I wanted to believe it was nothing

Hours later, I saw him

In the hospital room

With the thin, blue gown

We had seen so many times

On our own patients

His face was washed out

Like driftwood on a seashore

So tired, so stubbornly brave

That I burst into tears

Because I knew then

All those years

He had been

Invincible

I want to end with poems about what matters most: why we do what we do. The first is called “We save a life, but then.”

**We save a life, but then**

We want you to

Hear notes

Sad, wild, longing

See worlds

Bright, free, daring

Grab truth

Soft, rough, mottled

Taste love

Bold, pink, luscious

We save a life but then

It needs something to live for

What is it you live for?

The last two poems speak to our world now. I’ve emphasized climate change as the key issue of our time, something our Society must work on in ways small, like carbon offsets and reducing travel, and big, like getting into the science of how it affects the diseases we study, and working on advocacy to combat this most threatening of all issues to ourselves and those we care for and work for. Five, 10, and 20 years from now, we’ll be judged by what we did to save our planet, this beautiful world we live in and are a part of. We’ll also be judged by whether we were willing to be honest about our mistakes and weaknesses. This poem is called “The loon.”

**The loon**

Swimming back to the shore, I saw a loon

His black head raised, white feathers above

The brown and black of his breast

Placid as a decoy

Calling across the lake

As if trying out his voice

He did not sense me approaching

Slicing the water with my slow strokes

He continued his plum-toned calls to no one

Quiet dips of his black head into the water

Until, so close I thought I would alarm him

I splashed a few times

The loon jerked up his head

Like a cartoon lady who gathers her skirts and

Flees at the sight of a mouse

He paddle-skied frantically across the lake

Wings churning, feathered wheels under his chariot body

Searing the lake water beneath him

His calls desperate and cracked

Thirty feet away, he stopped

Smoothed his feathers

His calls, measured and regular again

Echoed across the lake

Woot-oooooo!

He dipped his head back into the water

Like a rebuke

Friend loon,

I understand you

I, too, must remain

Unperturbed

But let me tell you this:

In that one brief moment of fear

You were magnificent

I want to thank you again for honoring me with the job of president of this great Society and end with a poem called, “Let us not speak tonight of eternity.”

**Let us not speak tonight of eternity**

Let us not speak tonight of eternity

Instead, let us talk of

Blood-red cardinals swooping into fiery orange canopies

The anxious hammering of the red-bellied woodpecker

On the white siding

The smooth currents that lift the red-tailed hawk

To its roosting place atop the burnt brown hospital bricks

Sink into the beauty before you

Tender, treacherous

Delicate, cruel

Every little thing susceptible

And tell me your pledge to this sacred world

 Tell me *your* pledge to this sacred world.

 Thank you.

